# Racial/Ethnic Differences in Cardiometabolic Risk in a Community Sample of Sexual Minority Women

**DOI:** 10.1089/heq.2019.0024

**Published:** 2019-07-01

**Authors:** Billy A. Caceres, Cindy B. Veldhuis, Tonda L. Hughes

**Affiliations:** Columbia University School of Nursing, New York, New York.

**Keywords:** sexual minority, cardiovascular health, racial minority

## Abstract

**Purpose:** To examine the intersection of sexual identity and race/ethnicity on self-reported cardiometabolic risk in sexual minority women (SMW).

**Methods:** Data from the Chicago Health and Life Experiences of Women study were analyzed. Logistic regression models examined racial/ethnic differences in cardiometabolic risk (including obesity, hypertension, and diabetes) in SMW, accounting for psychosocial and behavioral factors. A variable accounting for the intersection of sexual identity and race/ethnicity was added to regression models (White lesbian women were the reference group).

**Results:** The analytic sample included 601 SMW (237 White, 219 Black, 145 Latina). Black (adjusted odds ratio [AOR] 2.96, 95% confidence interval [CI]=1.48–5.94) and Latina (AOR 2.30, 95% CI=1.18–4.48) SMW had higher rates of lifetime trauma than White SMW. Black SMW reported higher rates of obesity (AOR 3.05, 95% CI=1.91–4.88), hypertension (AOR 1.99, 95% CI=1.08–3.66), and diabetes (AOR 3.77, 95% CI=1.46–9.74) relative to White SMW. Intersectional analyses revealed that Black lesbian (AOR 2.94, 95% CI=1.74–4.97) and Black bisexual (AOR 3.43, 95% CI=1.69–6.96) women were more likely to be obese than White lesbian women. Black lesbian women also reported higher rates of hypertension (AOR 2.09, 95% CI=1.08–4.04) and diabetes (AOR 3.31, 95% CI=1.26–8.67) than White lesbian women. No differences in cardiometabolic risk were found between Latina and White SMW.

**Conclusion:** This study extends previous research on racial/ethnic differences in cardiometabolic risk among SMW. Prevention strategies are needed to reduce cardiometabolic risk in Black SMW. Findings highlight the need for cardiovascular disease research in SMW that incorporates longitudinal designs and objective measures.

## Introduction

Cardiovascular disease (CVD) is the leading cause of death and disability among women worldwide.^1^ Modifiable factors (psychosocial factors, smoking, physical activity, diet, alcohol use, obesity, hypertension, diabetes, and lipids) account for ∼94% of cardiometabolic risk in women.^2^ Although CVD disparities related to sex/gender and race/ethnicity are well documented,^3,4^ little is known about sexual identity differences in cardiometabolic risk. There is mounting evidence of higher cardiometabolic risk in sexual minority women (SMW; e.g., lesbian, bisexual) compared to heterosexual women. SMW have higher rates of poor mental health,^5–8^ tobacco use,^6,9–11^ heavy drinking,^6,12,13^ obesity,^8,14–18^ and hyperglycemia^15,19^ that may predispose them to CVD relative to heterosexual women.

In the United States, Black and Latina women have significantly elevated cardiometabolic risk compared with White women.^20,21^ Although racial/ethnic minority women report lower rates of tobacco use and heavy drinking than White women, they are less likely to meet physical activity guidelines.^20,22^ Further, the prevalence of obesity is highest among Black and Latina women in comparison to White women.^23^ Recent data suggest that the prevalence of hypertension and diabetes is highest among Black women, whereas Latina women only have higher rates of diabetes relative to White women.^20^ The death rate attributed to hypertension is highest among Black women^24^ and Black women have higher rates of incident CVD than White women, regardless of their baseline cardiometabolic risk.^25^

Despite reasons to hypothesize that cardiometabolic risk is elevated in SMW, few studies have examined racial/ethnic differences in this population. White and Black SMW are more likely to be overweight than heterosexual women of the same race/ethnicity.^26^ Several studies report higher rates of obesity among Black^27–29^ and Latina^27,28^ SMW compared with heterosexual women of the same race/ethnicity. White, Black, and Latina SMW each report higher rates of current tobacco use than their heterosexual counterparts.^10,30,31^ White and Black SMW also report higher rates of alcohol consumption and history of stroke than heterosexual women of the same race/ethnicity.^31^

Racial/ethnic differences have been observed when comparing cardiometabolic risk between White SMW and SMW of color. Latina lesbian women report higher rates of obesity and diabetes than White lesbian women, and Black bisexual women are more likely than White bisexual women to report being obese.^32^ Moreover, Black SMW are more likely to report lower fruit/vegetable intake and physical activity, but higher rates of elevated body mass index (BMI), hypertension, and diabetes than White SMW.^29^ Additional research investigating heterogeneity in cardiometabolic risk among SMW is needed to determine whether the racial/ethnic disparities observed in the general population are present in this group.

An intersectional approach supports the investigation of how multiple intersecting forms of oppression may be associated with health outcomes. Much of this research has taken an additive approach by focusing on the main effects of sexual identity or race/ethnicity, separately, which ignores the potential interactions between stigmatized identities. Richardson and Brown^33^ used an intersectional approach to test the interaction of race/ethnicity and sex/gender on hypertension risk. They showed that the interaction of race/ethnicity and sex/gender had a greater influence on hypertension risk than either alone. Black and Latina women had higher hypertension risk than White women and Black and Latino men. That evidence supports the use of intersectional approaches to investigate cardiometabolic risk in vulnerable women.

Using data from a large, diverse sample of SMW, this study builds on previous research. This study uses an intersectional approach to examine cardiometabolic risk in a community sample of cisgender (non-transgender) SMW. Given the dearth of evidence on the intersection of sexual identity and race/ethnicity on cardiometabolic risk in women, we conducted cross-sectional analyses of data from the Chicago Health and Life Experiences of Women (CHLEW) study, a longitudinal study of health and well-being of SMW. CHLEW is unique because relatively few studies of SMWs health have adequate sample sizes to test how race/ethnicity and sexual identity interact to influence health outcomes.^34–36^

The purpose of this study was to examine the intersection of sexual identity and race/ethnicity on cardiometabolic risk factors accounting for psychosocial factors and health behaviors in a community sample of SMW.

## Methods

### Sample

The CHLEW study (*N*=723) is a 19-year longitudinal study, funded by the National Institute on Alcohol Abuse and Alcoholism, that focuses on risk and protective factors associated with alcohol use and the health of cisgender SMW. Since 2000, three waves of data have been collected and a fourth wave is currently underway. Wave 1 of the CHLEW (2000–2001) included a convenience sample of 447 English-speaking lesbian women older than the age of 18 recruited from the Chicago metropolitan area. Information about the original sample and sampling methods are reported elsewhere.^37^ Wave 2 (2004–2005) conducted follow-up interviews with 384 women (86%) of the Wave 1 cohort. CHLEW Wave 3 (2010–2012) retained 353 women (79%) from the original cohort and added a supplemental sample (*n*=370) of bisexual women, younger (18–25 years) women, and Black and Latina women.^38^

We analyzed data from CHLEW Wave 3 because this wave includes the most diverse sample to support an intersectional approach for assessing cardiometabolic risk in SMW. The CHLEW study was approved by the Principal Investigator's home institution.

### Eligibility criteria

Women who identified as mostly or only heterosexual/straight (*n*=14) or another sexual identity (*n*=13) were excluded from this study. Participants who identified their race as “other” (*n*=24) were excluded due to sample size constraints for intersectional analyses. Women with missing data for the remaining study variables were also excluded (*n*=72). Women who identified as lesbian (*n*=352) and mostly lesbian (*n*=100) were combined into one category and labeled lesbian women. A total of 149 bisexual women were included.

### Measures

#### Sexual identity

Sexual identity was assessed by asking women: “Recognizing that sexual identity is only one part of your identity how do you define your sexual identity? Would you say that you are: only lesbian/gay, mostly lesbian/gay, bisexual, mostly heterosexual/straight, only heterosexual/straight?”

#### Demographic characteristics

Race/ethnicity was defined as White, Black, and Latina. Demographic characteristics included age (categorized as 18–30, 31–40, 41–50, and 51–75), household income (<$20,000, $20,000–39,999, $40,000–74,999, and ≥ $75,000), education (less than high school, high school, some college, college graduate, and graduate school), employment (full-time, part-time, unemployed-looking for work, and unemployed-not looking for work), relationship status (committed-cohabitating, committed-not cohabitating, and single), and health insurance coverage (yes/no).

#### Psychosocial factors

*Lifetime depression* was assessed with criteria from the National Institute of Mental Health Diagnostic Interview Schedule.^39^ A depressive episode was defined as the persistence of four or more of depressive symptoms for at least 2 weeks, in addition to feeling sad, blue, or depressed or by loss of interest or pleasure in things usually cared about and was dichotomized as any depressive episode versus none.

*Social support* was assessed with the Multidimensional Scale of Perceived Social Support (MSPSS), a 12-item measure of social support with a total possible score of 84.^40^ Total scores were divided by 12 and categorized social support based on established cutoffs: low (1.0–2.9), moderate (3.0–5.0), or high (5.1–7.0).^41^ Since only 15 (2.5%) participants reported low social support, we dichotomized social support by combining the categories of low and moderate (32.5%). We examined low/moderate versus high social support (65.0%) in regression analyses.

*Perceived discrimination* included six questions about experiences of discrimination in the past 12 months.^42^ A score of “1” was assigned for each experience reported, and the total number of experiences of discrimination was then summed (0–6). We dichotomized discrimination so that a score of “0” indicated no experiences of discrimination and a score of “1” indicated at least one experience of discrimination.

Previous analyses of CHLEW data suggest that lifetime trauma is associated with higher rates of obesity, hypertension, and diabetes in SMW.^43^ Therefore, we assessed *lifetime trauma* by combining reports of childhood and adulthood trauma based on established methods.^44–46^

Childhood trauma included physical abuse, sexual abuse, and parental neglect before the age of 18. Childhood physical abuse was assessed with the following item: “Do you feel that you were physically abused by your parents or other family members when you were growing up?” and was coded dichotomously (1=Yes; 0=No). Childhood sexual abuse was measured following established criteria.^47^ Responses were coded as “1” indicating presence and “0” indicating absence of childhood sexual abuse. To assess parental neglect, participants were asked whether they believed their basic needs (such as food, shelter, etc.) were neglected when they were growing up (1=Yes; 0=No).

Adulthood trauma included physical assault, sexual assault, and intimate partner violence (IPV) after the age of 18. For adult physical assault, participants were asked two questions assessing whether someone (other than their partner) had ever attacked them with or without a weapon with the intent to kill or seriously injure them. Participants who responded affirmatively to one or both questions were categorized as having experienced adult physical assault (1=Yes; 0=No). To assess adult sexual assault, participants were asked whether “since the age of 18 was there a time when you experienced any unwanted/forced sexual activity?” Responses were coded dichotomously (1=Yes; 0=No).

IPV was assessed by asking participants whether a partner had ever sexually assaulted them or whether a recent partner ever “threw something at you, pushed you, or hit you?” or “threatened to kill you, with a weapon or in some other way?” Three forms of IPV (sexual assault, physical assault, and threat of harm) were summed and dichotomized (1=Any IPV; 0=No IPV).

A cumulative lifetime trauma score (0–6) was created based on the sum of childhood and adulthood trauma. A score of “0” indicated no report of lifetime trauma, whereas a score of “6” indicated presence of all forms of trauma assessed. We dichotomized lifetime trauma (1=Yes; 0=No).

#### Health behaviors

Participants were asked whether they currently smoked cigarettes (1=Yes; 0=No). Heavy episodic drinking was coded as a binary variable by using a question that asked whether in the past year the participant had ever consumed six or more drinks in a day (1=Yes; 0=No).^48^ Overeating was measured by asking participants whether in the past 3 months they had consumed what would be considered by others to be a large amount of food in a short period (1=Yes; 0=No).

#### Cardiometabolic risk

BMI (kg/m^2^) was calculated by using self-reported weight and height and classified as obesity if greater than or equal to 30.0 kg/m^2^.^49^ Also, we assessed whether a health care provider had ever diagnosed participants with hypertension and/or diabetes based on participant self-report (yes/no).

### Statistical analysis

Data analysis was performed in Stata, version 15. White women (the largest group) were the reference group for all analyses. We conducted chi-square tests and Fisher exact tests (for education and social support) to assess racial/ethnic differences across study variables. White women were compared separately with Black and Latina women. A significance level of *p*<0.05 was predetermined.

We used multiple logistic regression models to examine racial/ethnic differences in psychosocial factors and health behaviors adjusted for demographic characteristics. Next, we ran multiple logistic regression models to examine racial/ethnic differences in cardiometabolic risk factors (obesity, hypertension, and diabetes). Model 1 was unadjusted, Model 2 added adjustment for demographic characteristics, and Model 3 added psychosocial factors and health behaviors. Since obesity is a risk factor for hypertension and diabetes in adults,^50–54^ Model 3 for hypertension and diabetes was also adjusted for obesity.

To examine the intersection of sexual identity and race/ethnicity, we created a six-category interaction variable for sexual identity (lesbian and bisexual) and race/ethnicity (White, Black, and Latina). This interaction variable was then added to logistic regression models to assess the interaction of sexual identity and race/ethnicity on cardiometabolic risk with White lesbian women as the reference group. We were unable to compare racial/ethnic minority bisexual women with White bisexual women in intersectional analyses due to limited sub-sample sizes (*n*=54 White, 60 Black, and 36 Latina).

## Results

Table 1 presents descriptive statistics. The analytic sample included 601 SMW (452 lesbian and 149 bisexual; 237 White, 219 Black, and 145 Latina). Compared with White SMW, Black SMW were younger (*p*=0.03), reported lower household income (*p*<0.001), had lower educational attainment (*p*<0.001), were less likely to be currently employed (*p*<0.001), more likely to be single (*p*<0.001), and less likely to have health care insurance (*p*<0.001). Latinas were younger (*p*<0.001), had lower educational attainment (*p*<0.001), were more likely to be single (*p*<0.01), and less likely to have health care insurance (*p*<0.001). Latinas were also more likely than White women to report that they were unemployed but looking for work (*p*<0.01).

**Table 1.** Descriptive Statistics for Sexual Minority Women by Race/Ethnicity (*N*=601)

	Total sample (*N*=601), *n* (%)	White SMW (*N*=236), *n* (%)	Black SMW (*N*=220), *n* (%)	*p*-Value Black vs. White SMW	Latina SMW (*N*=145), *n* (%)	*p*-Value Latina vs. White SMW
Demographic characteristics						
Sexual identity				0.28		0.66
Lesbian	451 (75.0)	182 (77.1)	160 (72.7)		109 (75.2)	
Bisexual	150 (25.0)	54 (22.9)	60 (27.3)		36 (24.8)	
Age				0.02^*^		<0.001^*^
18–30	186 (31.0)	64 (27.4)	60 (27.3)		62 (42.8)	
31–40	130 (21.6)	48 (20.3)	47 (21.4)		35 (24.1)	
41–50	120 (20.0)	38 (16.0)	57 (25.9)		25 (17.2)	
51–75	165 (27.4)	86 (36.3)	56 (25.4)		23 (15.9)	
Household income				<0.001^*^		0.07
<$20,000	195 (32.4)	50 (21.2)	109 (49.6)		36 (24.8)	
$20,000–39,999	117 (19.5)	47 (19.9)	46 (20.9)		24 (16.6)	
$40,000–74,999	143 (23.8)	52 (22.0)	45 (20.5)		46 (31.7)	
≥$75,000	146 (24.3)	87 (36.9)	20 (9.0)		39 (26.9)	
Education				<0.001^*^		<0.001^*^
Less than high school	40 (6.7)	2 (0.9)	28 (12.7)		10 (6.9)	
High school	76 (12.7)	8 (3.4)	49 (22.3)		19 (13.1)	
Some college	186 (30.9)	52 (22.0)	81 (36.8)		53 (36.6)	
College graduate	130 (21.6)	68 (28.8)	33 (15.0)		29 (20.0)	
Graduate school	169 (28.1)	106 (44.9)	29 (13.2)		34 (23.4)	
Employment				<0.001^*^		0.01^*^
Full-time	270 (45.0)	118 (50.0)	75 (34.1)		77 (53.1)	
Part-time	142 (23.6)	59 (25.0)	50 (22.7)		33 (22.8)	
Unemployed, looking	91 (15.1)	17 (7.2)	51 (23.2)		23 (15.9)	
Unemployed, not looking	98 (16.3)	42 (17.8)	44 (20.0)		12 (8.2)	
Relationship status				<0.001^*^		<0.01^*^
Committed, cohabitating	233 (38.8)	119 (50.4)	63 (28.6)		51 (35.2)	
Committed, not cohabitating	134 (22.3)	40 (17.0)	58 (26.4)		36 (24.8)	
Single	234 (38.9)	77 (32.6)	99 (45.0)		58 (40.0)	
Health insurance	428 (71.2)	195 (82.6)	135 (61.4)	<0.001^*^	98 (67.6)	<0.001^*^
Psychosocial factors						
Lifetime depression	350 (58.2)	163 (69.1)	101 (45.9)	<0.001^*^	86 (59.3)	0.06
Perceived social support				<0.001^*^		0.52
Low	15 (2.5)	5 (2.1)	4 (1.8)		6 (4.1)	
Moderate	195 (32.5)	61 (25.9)	97 (44.1)		37 (25.5)	
High	391 (65.0)	170 (72.0)	119 (54.1)		102 (70.3)	
Discrimination in past year	288 (47.9)	109 (46.2)	112 (50.9)	0.31	67 (46.2)	0.99
Any lifetime trauma	522 (86.9)	186 (78.8)	206 (93.6)	<0.001^*^	130 (89.7)	0.01^*^
Health behaviors						
Current tobacco use	182 (30.3)	46 (19.5)	98 (44.6)	<0.001^*^	38 (26.2)	0.13
Binge drinking (past year)	237 (39.4)	83 (35.2)	83 (37.7)	0.57	71 (49.0)	<0.01^*^
Overeating (past 3 months)	102 (17.0)	39 (16.5)	37 (16.8)	0.93	26 (17.9)	0.72
Cardiometabolic risk						
Obesity (BMI ≥30.0 kg/m^2^)	226 (37.6)	60 (25.4)	117 (53.2)	<0.001^*^	49 (33.8)	0.08
Hypertension	113 (18.8)	33 (14.0)	62 (28.2)	<0.001^*^	18 (12.4)	0.66
Diabetes	46 (7.7)	9 (3.8)	30 (13.6)	<0.001^*^	7 (4.8)	0.63

Reference group=White SMW.

^*^ *p*<0.05.

BMI, body mass index; SMW, sexual minority women.

Black women reported lower social support (*p*<0.001). Black and Latina women had higher rates of exposure to lifetime depression and trauma than White SMW. Black women reported higher rates of current tobacco use (*p*<0.001), and Black (*p*=0.02) and Latina (*p*<0.01) women were more likely to report binge drinking.

Table 2 presents results of logistic regression analyses for racial/ethnic differences in psychosocial factors and health behaviors. Black women were less likely to report lifetime depression than White women (adjusted odds ratio [AOR] 0.39, 95% confidence interval [CI]=0.25–0.60). In fully adjusted models, Black (AOR 2.89, 95% CI=1.44–5.81) and Latina (AOR 2.27, 95% CI=1.17–4.40) women were more likely to report any lifetime trauma compared with White women. SMW of color reported higher rates of binge drinking (Black OR 1.92, 95% CI=1.16–3.18; Latina OR 1.95, 95% CI=1.12–3.40) than White women in unadjusted analyses; however, these differences were attenuated after covariate adjustment.

**Table 2.** Racial/Ethnic Differences in Psychosocial Factors and Health Behaviors Among Sexual Minority Women (*N*=601)

	Model 1, OR 95% CI	Model 2, AOR 95% CI
Psychosocial factors		
Lifetime depression		
White	Ref	Ref
Black	0.38 (0.26–0.56)^*^	0.40 (0.26–0.62)^*^
Latina	0.65 (0.42–0.99)^*^	0.71 (0.45–1.11)
High social support		
White	Ref	Ref
Black	0.46 (0.31–0.67)^*^	0.66 (0.43–1.03)
Latina	0.92 (0.58–1.45)	1.04 (0.63–1.71)
Discrimination in past year		
White	Ref	Ref
Black	1.21 (0.84–1.75)	1.13 (0.74–1.73)
Latina	1.00 (0.66–1.52)	0.79 (0.51–1.25)
Any lifetime trauma		
White	Ref	Ref
Black	3.96 (2.12–7.39)^*^	2.96 (1.48–5.94)^*^
Latina	2.33 (1.25–4.33)^*^	2.30 (1.18–4.48)^*^
Health behaviors		
Current tobacco use		
White	Ref	Ref
Black	3.32 (2.19–5.04)^*^	1.35 (0.82–2.22)
Latina	1.47 (0.90–2.40)	0.73 (0.41–1.29)
Binge drinking		
White	Ref	Ref
Black	1.12 (0.76–1.64)	0.85 (0.53–1.34)
Latina	1.77 (1.16–2.70)^*^	1.10 (0.69–1.77)
Overeating		
White	Ref	Ref
Black	1.02 (0.62–1.67)	0.81 (0.46–1.42)
Latina	1.10 (0.64–1.91)	0.78 (0.43–1.40)

Model 1 unadjusted; Model 2 adjusted for demographic characteristics.

^*^ *p*<0.05.

AOR, adjusted odds ratio; CI, confidence interval.

Table 3 shows results of logistic regression analyses for racial/ethnic differences in cardiometabolic risk. No differences in cardiometabolic risk were found between Latina and White SMW. In fully adjusted models, Black SMW reported higher rates of obesity (AOR 3.05, 95% CI=1.91–4.88), hypertension (AOR 1.99, 95% CI=1.08–3.66), and diabetes (AOR 3.77, 95% CI=1.46–9.74) relative to their White counterparts.

**Table 3.** Racial/Ethnic Differences in Cardiometabolic Risk Among Sexual Minority Women (*N*=601)

	Model 1, OR 95% CI	Model 2, AOR 95% CI	Model 3, AOR 95% CI^a^
Cardiometabolic risk			
Obesity			
White	Ref	Ref	Ref
Black	3.33 (2.23–4.95)^*^	2.85 (1.82–4.45)^*^	2.94 (1.85–4.70)^*^
Latina	1.50 (0.95–2.35)	1.51 (0.93–2.46)	0.90 (0.89–2.41)
Hypertension			
White	Ref	Ref	Ref
Black	2.41 (1.51–3.87)^*^	2.23 (1.27–3.95)^*^	1.97 (1.07–3.63)^*^
Latina	0.87 (0.47–1.61)	1.25 (0.63–2.48)	1.16 (0.57–2.36)
Diabetes			
White	Ref	Ref	Ref
Black	3.98 (1.84–8.60)^*^	4.79 (1.98–11.63)^*^	3.81 (1.47–9.84)^*^
Latina	1.28 (0.47–3.51)	2.19 (0.74–6.42)	1.88 (0.60–5.85)

Model 1 unadjusted; Model 2 adjusted for demographic characteristics; Model 3 adjusted for demographic characteristics, psychosocial factors, and health behaviors.

^a^ Hypertension and diabetes also adjusted for obesity in Model 3.

^*^ *p*<0.05.

Table 4 presents results of the logistic regression analyses examining the intersection of sexual identity and race/ethnicity on cardiometabolic risk. In fully adjusted models, Black lesbian (AOR 2.94, 95% CI=1.74–4.97) and Black bisexual (AOR 3.43, 95% CI=1.69–6.96) women were more likely to be obese than White lesbian women. Black lesbian women were more likely to report a history of hypertension (AOR 2.09, 95% CI=1.08–4.04) and diabetes (AOR 3.31, 95% CI=1.26–8.67) than White lesbian women. Intersectional analyses for diabetes produced empty cells as none of the White bisexual participants had diabetes.

**Table 4.** Intersection of Sexual Identity and Race/Ethnicity on Cardiometabolic Risk (*N*=601)

Cardiometabolic risk	Model 1, OR 95% CI	Model 2, AOR 95% CI	Model 3, AOR 95% CI^a^
Obesity			
White lesbian	Ref	Ref	Ref
Black lesbian	3.24 (2.06–5.10)^*^	2.77 (1.65–4.65)^*^	2.85 (1.69–4.81)^*^
Latina lesbian	1.56 (0.93–2.60)	1.65 (0.95–2.86)	1.61 (0.92–2.82)
White bisexual	0.80 (0.39–1.64)	1.08 (0.50–2.36)	1.04 (0.48–2.28)
Black bisexual	2.98 (1.63–5.46)^*^	3.02 (1.51–6.05)^*^	3.20 (1.59–6.44)^*^
Latina bisexual	1.07 (0.48–2.39)	1.06 (0.44–2.56)	1.08 (0.45–2.62)
Hypertension			
White lesbian	Ref	Ref	Ref
Black lesbian	2.37 (1.39.99)^*^	2.38 (1.26–4.49)^*^	2.06 (1.07–3.97)^*^
Latina lesbian	0.88 (0.45–1.73)	1.36 (0.64–2.90)	1.29 (0.59–2.80)
White bisexual	0.56 (0.21–1.53)	1.02 (0.34–3.07)	1.14 (0.37–3.52)
Black bisexual	1.67 (0.81–3.44)	2.14 (0.88–5.23)	1.75 (0.68–4.50)
Latina bisexual	0.50 (0.14–1.74)	0.77 (0.19–3.14)	0.78 (0.19–3.28)
Diabetes			
White lesbian	Ref	Ref	Ref
Black lesbian	3.57 (1.61–7.88)^*^	3.99 (1.56–10.21)^*^	3.31 (1.26–8.70)^*^
Latina lesbian	1.12 (0.39–3.23)	1.87 (0.59–5.86)	1.64 (0.50–5.44)
White bisexual	Empty cell	Empty cell	Empty cell
Black bisexual	1.75 (0.56–5.43)	2.38 (0.61–9.36)	1.92 (0.45–8.18)
Latina bisexual	0.55 (0.07–4.47)	0.98 (0.10–9.66)	0.89 (0.09–9.03)

Model 1 unadjusted; Model 2 adjusted for demographic characteristics; Model 3 adjusted for demographic characteristics, psychosocial factors, and health behaviors.

^a^ Hypertension and diabetes were also adjusted for obesity in Model 3.

^*^ *p*<0.05.

## Discussion

This study builds on previous work examining racial/ethnic disparities in cardiometabolic risk^29,32^ in a diverse sample of SMW. Although Molina et al. had a larger overall sample size, there were no Latinas included in that study and the number of Black SMW (*n*=75) was less than half of that in this study (*n*=222).^29^

The higher rates of lifetime depression and trauma reported by SMW of color are consistent with a recent analysis of CHLEW data.^46^ No racial/ethnic differences in health behaviors were identified. With a few exceptions,^55^ there is a paucity of research examining racial/ethnic differences in psychosocial and behavioral risk factors for CVD in SMW.^6^ Future studies should use intersectional approaches to examine which subgroups of SMW are most at risk for CVD.

We found that Black SMW had higher rates of obesity, hypertension, and diabetes than White SMW. These findings are consistent with national estimates indicating Black women overall have higher cardiometabolic risk than White women.^20,23^ Our findings also corroborate evidence documenting disparities in cardiometabolic risk between Black and White SMW.^29^

Although Molina et al.^29^ found higher rates of elevated BMI, hypertension, and diabetes in Black SMW compared with White SMW, they did not examine lesbian and bisexual women separately. This is a noted strength of this study as intersectional analyses revealed that Black lesbian women reported higher rates of obesity, hypertension, and diabetes relative to White lesbian women, whereas Black bisexual women had only higher rates of obesity. Given that no White bisexual participants reported a history of diabetes, we were unable to examine differences between White bisexual and White lesbian women for this risk factor.

Analyses of cross-sectional data from the National Epidemiologic Survey on Alcohol and Related Conditions found that major depressive disorder and generalized anxiety disorder partially mediated the association between sexual identity and CVD prevalence.^56^ Although cross-sectional analyses tend to overestimate the influence of potential mediators,^57^ there is a need for future research that examines potential mediators of the association of sexual identity and cardiometabolic risk in SMW. In particular, more prospective studies that examine how mental health conditions and minority stressors (e.g., discrimination, victimization, and expectations of rejection) potentially mediate the associations between race/ethnicity and cardiometabolic risk in SMW are warranted.

It could be argued that the stress from having multiple stigmatized identities (e.g., being a racial/ethnic minority, a sexual minority, and a woman) may increase the likelihood of experiencing multiple forms of acute and chronic stressors related to those identities, which, in turn, may synergistically increase cardiometabolic risk. Findings from this study have important implications for cardiometabolic risk reduction among SMW. Clinicians should be educated about the higher rates of CVD risk factors observed in Black SMW.

In particular, it is important that clinicians understand that subgroups within the larger group of SMW may be at differential risk for CVD. Increased knowledge of racial/ethnic differences is crucial for the development of much needed culturally tailored interventions for cardiometabolic risk reduction in SMW.

### Limitations

Given that ∼50% of participants were added to the CHLEW study in Wave 3, we were unable to conduct longitudinal analyses. Thus, causality cannot be inferred from these findings. As psychosocial factors and health behaviors are posited to potentially mediate the association between sexual identity and race/ethnicity with cardiometabolic risk, there is a need for prospective studies that permit assessment of the temporality of these risk factors.

Selection bias is a possible limitation related to attrition in the CHLEW study. We conducted analyses to examine predictors of attrition in the CHLEW study and found that the only significant predictor of attrition from Wave 1 to Wave 2 was lower educational attainment (*p*<0.05). Predictors of attrition from Wave 2 to Wave 3 were having a history of childhood sexual abuse (*p*<0.05) and having one or more children living at home (*p*<0.05). There were no sexual identity or racial/ethnic differences in attrition in the CHLEW study.

Given the recognized link between lower educational attainment^58–62^ and childhood sexual abuse^63–66^ with cardiometabolic risk and incident CVD, we believe that SMW from the original CHLEW sample that remained in Wave 3 may actually have lower cardiometabolic risk than women who dropped out. Therefore, this study likely underestimates the prevalence of cardiometabolic risk factors in SMW.

Further, we were unable to examine whether the higher rates of obesity, hypertension, and diabetes in Black SMW differ from their heterosexual counterparts of the same race/ethnicity. This is an important area for future research as recent population-based data indicate that only White lesbian women report higher rates of obesity than their heterosexual peers.^32^

Also, data on cardiometabolic risk factors were obtained from participant self-report. Previous research indicates that both sexual minority and heterosexual women underreport their BMI.^67^ Several validation studies indicate that there is moderate-to-high concordance between self-report of hypertension and diabetes with objective measures (such as data from medical records and objective assessments).^68–72^ However, the validity of self-reported hypertension and diabetes varies by age, socioeconomic status, education, and health care use.^68,69^

The use of objective measurements of cardiometabolic risk factors is preferred. As a few studies have incorporated objective measures of cardiometabolic risk factors to study CVD in SMW, this is an important area to consider in future research.^6,73^ Although the use of objective measures would have strengthened our findings, this study represents an important contribution to research on CVD in SMW.

Intersectional analyses were limited, as no White bisexual participants reported a history of diabetes and a few participants reported a history of diabetes (*n*=46). The smaller sample of bisexual participants also limited statistical power to compare racial/ethnic minority bisexual women with White bisexual women. Our analyses should be replicated with larger samples that include more women with a history of diabetes and more bisexual women.

## Conclusion

These findings contribute to the nascent body of research examining heterogeneity in cardiometabolic risk among diverse SMW. The higher rates of cardiometabolic risk factors observed in Black SMW, particularly Black lesbian women, suggest a need for prevention strategies to reduce risk in this group. Our findings highlight areas for future research, including the need for longitudinal analyses and inclusion of objectively measured cardiometabolic risk factors.

## Abbreviations Used

AOR: adjusted odds ratio
BMI: body mass index
CHLEW: Chicago Health and Life Experiences of Women
CI: confidence interval
CVD: cardiovascular disease
IPV: intimate partner violence
MSPSS: Multidimensional Scale of Perceived Social Support
SMW: sexual minority women
